# Thermal carbonization in nanoscale reactors: controlled formation of carbon nanodots inside porous CaCO_3_ microparticles

**DOI:** 10.1038/s41598-018-27488-w

**Published:** 2018-06-20

**Authors:** Anna V. Vostrikova, Ekaterina S. Prikhozhdenko, Oksana A. Mayorova, Irina Yu. Goryacheva, Nadezda V. Tarakina, Gleb B. Sukhorukov, Andrei V. Sapelkin

**Affiliations:** 1 Saratov State University, 83 Astrakhanskaya Street, Saratov, 410012 Russia; 2 School of Engineering and Materials Science, Queen Mary University of London, Mile End Road, London, E1 4NS UK; 3 School of Physics and Astronomy, Queen Mary University of London, Mile End Road, London, E1 4NS UK

## Abstract

Synthesis of carbon nanodots (CNDs) in confined geometry via incorporation of dextran sulphate into pores of CaCO_3_ microparticles is demonstrated. The preparation process included three steps: co-precipitation of solutions of inorganic salts and carbon source, thermal treatment and CaCO_3_ matrix removal. We show that geometric constraints can be used to precisely control the amount of source material and to avoid formation of large carbon particles. Analysis of TEM data shows particle size of ~3.7 nm with narrow size distribution. Furthermore, we found that variation in pore morphology has a clear effect on CNDs structure and optical properties. CNDs with graphene oxide like structure were obtained in the nanoporous outer shell layer of CaCO_3_ microparticles, while less ordered CNDs with the evidence of complex disordered carbons were extracted from the inner microcavity. These results suggest that confined volume synthesis route in CaCO3 nanopores can be used to precisely control the structure and optical properties of CNDs.

## Introduction

Light emitting carbon nanodots (CNDs) have recently emerged as a new family of low dimensional nanocarbon materials. Compared to the more conventional light emitting quantum dots (e.g. CdSe, CdS, Si and Ge etc.), CNDs have clear advantages^1–4^ such as low environmental impact, low cytotoxicity, excitation-dependent emission wavelength, excellent biocompatibility, tunable surface functionalities, stability under ambient conditions. These appealing properties of CNDs suggest great opportunities for applications ranging from consumer electronics^5^, to light harvesting^6–8^ and biological cell imaging^7^. As a consequence, the field has been growing rapidly with thousands of articles published over the last few years. One of the major approaches to synthesis of CNDs is “bottom-up” preparation of structured materials via controlled assembly of atoms and molecules. This approach allows the synthesis of CNDs from a wide variety of molecular precursors - mainly carbon sources with diverse properties and composition. Vast majority of the synthesis routes involve high-temperature treatment options: pyrolysis^9^, ultrasonic^4,10–12^ or microwave radiation^13–18^, solvotermal^19,20^ and hydrothermal carbonization methods^21–24^. The latter route being particularly wide-spread due to its relative simplicity. The main problems arising in the synthesis of CNDs are non-uniformity in morphology and size distribution, formation of by-products, in particular, large carbon particles^8,25–29^. The heterogeneous nature of the reaction products is a consequence of relatively poor control over the synthesis environment since the molecular diffusion, temperature fluctuations and particle growth conditions cannot be effectively controlled on the nanoscale level in large volume reactors.

Micro- and nano-porous structures are a promising type of synthesis environment because of a possibility to perform reaction in a restricted volume with precisely controlled amount of reagents. There are a number of advantages in using porous structures as reaction volumes such as varying the size and morphology of the pores and access to a variety of matrixes (including metals, silica, inorganic and organic particles^30–32^ and polymers^33,34^). Furthermore, pore geometry may have a significant effect on carbon-based systems in particular by influencing preference for configurations with sp, sp^2^ and sp^3^ hybridizations (bonding) and hence can be used to control the atomic structure and functionality of the final product.

Porous structures have already been used for preparation of CNDs utilizing, for example, impregnation of mesoporous silica^35^. However, there are a number of significant disadvantages using mesoporous silica as a reaction volume which may restrict their commercial potential: the complexity of sample preparation within the mesoporous silica, complexity of removing the host material (etching with concentrated HF^36^ or NaOH^37^), and the undefined yield of the reaction product because of distribution of carbon source between pores and bulk solution. The impregnation is also associated with large losses of carbon material when only an impregnating solution inside the pores takes part in CNDs formation^36^.

At the same time, polymeric systems^38^ have already been used as a synthesis template while CaCO_3_ microparticles currently attract a lot of attention due to their ability to encapsulate various substances, controlled permeability, high surface-to-volume ratio and sufficient thermal stability. In this context, polycrystalline vaterite particles have a convenient spherical shape, developed surface and high porosity. The diameter of CaCO_3_ particles is typically from 1 to 6 μm^39^, the pore size is in the range of 20–70 nm and they are extensively used in engineering and bioengineering, chemical technology and are already compatible with the commercial manufacturing processes^40–43^. Besides, they are non-toxic and suitable for drug delivery systems development^44^. A significant advantage of these microreactors is the possibility of their removal under relatively mild conditions, using a solution of ethylenediaminetetraacetic acid^45^ or hydrochloric acid^46^.

In this work, we report a controlled formation of hydrophilic CNDs in pores of CaCO_3_ microparticles as a result of co-precipitation of inorganic salts (CaCl_2_ and Na_2_CO_3_) and sodium dextran sulfate (DS) as a carbon source, followed by thermal treatment. DS was chosen as carbon source because it is a non-toxic natural polymer, based on anhydroglucose and is routinely used for the selective precipitation of lipoproteins. We show that synthesis in a restricted volume has a clear effect on the structure and light emission in CNDs.

## Results and Discussion

**Figure 1:**
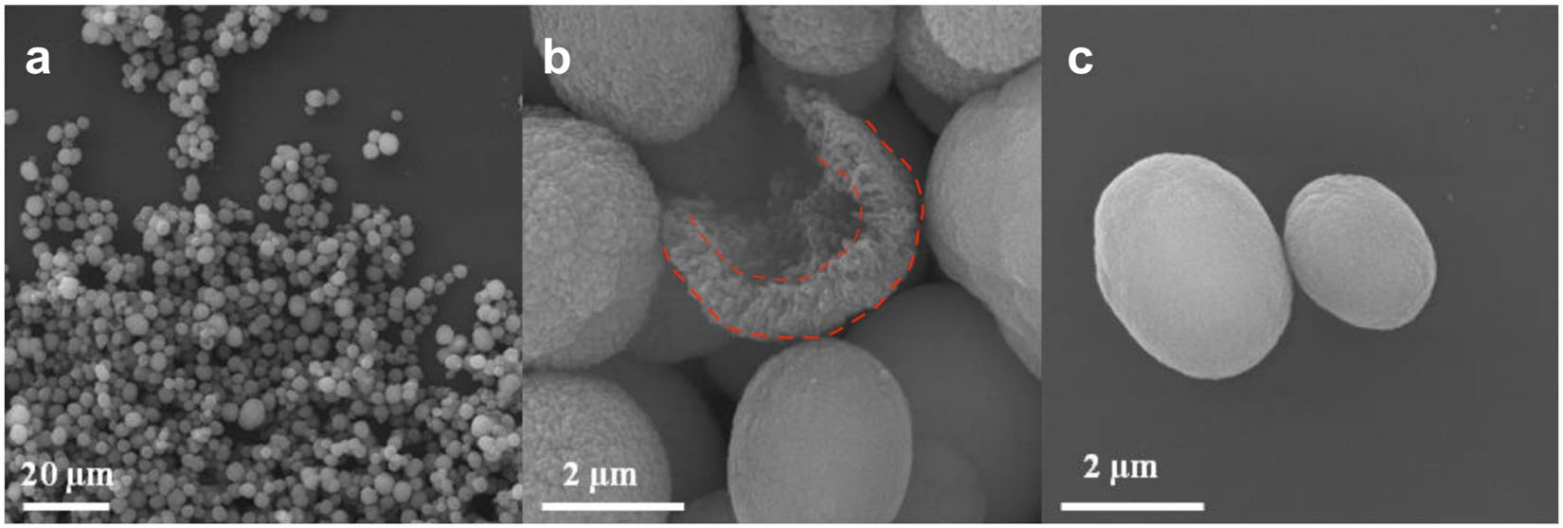
SEM images of CaCO_3_ microparticles (**a**), and of a broken particle (**b**) (with the shell structure outlined by the dashed red lines); the size and the surface morphology of DS-loaded CaCO_3_ microparticles is also shown (**c**).

CaCO_3_ microparticles with a spherical shape, porous structure and narrow size distribution (3–4 µm, Fig. 1a) were manufactured and used as the templates for CNDs synthesis. The cross-section image (Fig. 1b) further confirms the porous structure of the microparticles. One can also clearly see frequently observed^47^ shell-like structure (see broken red lines in Fig. 1b) of the CaCO_3_ microparticles which makes it possible to vary controllably synthesis conditions (i.e. degree of confinement of the ingredients) across the volume. The lower surface roughness of DS-loaded CaCO_3_ microparticles (CaCO_3_–DS, Fig. 1c) reflects presence of DS in CaCO_3_ microparticles and indirectly corroborates DS co-precipitation within CaCO_3_. This is further confirmed by confocal fluorescence imaging that shows presence of the light emitting material throughout the volume and the surface of CaCO_3_ microparticles (Fig. S1).

After thermal treatment CaCO_3_–DS microparticles colour has changed from white to brown while no soot was formed (Fig. S2). We examined photoluminescence (PL) from CNDs obtained in CaCO_3_ at various DS concentrations (2, 5 and 10 mg/ml) and did not observe significant differences between the spectra for the corresponding fractions at a given excitation wavelength (Fig. S3). Consequently, we only carried out further detailed analysis of the samples with initial DS concentration of 2 mg/ml. We also performed hydrothermal treatment of DS solutions (under identical temperature conditions) for reference where outcomes were clearly different: colour has changed to the dark-brown, soot was formed, while amount of soot (Fig. S4) and light emission (Fig. S5) were dependent on DS concentration.

**Figure 2:**
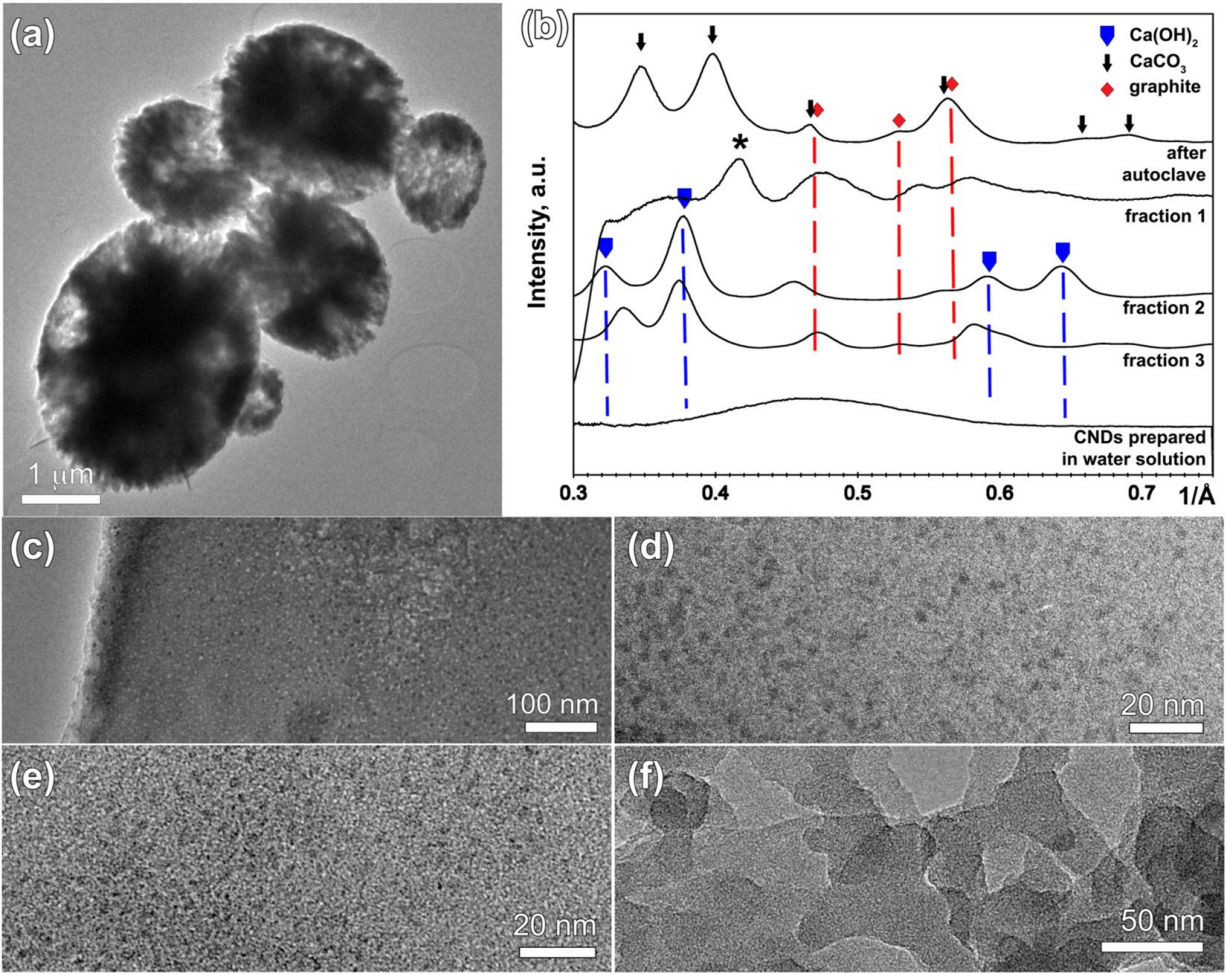
TEM images of (**a**) CaCO3-DS microcapsules (DS initial concentration 2 mg/ml); (**b**) azimuthal average profiles of SAED patterns collected from microcapsules; TEM images obtained from (**c**) fraction 1, (**d**) fraction 2, (**e**) fraction 3 and (**f**) from samples synthesized in water solution.

Following the synthesis, CNDs were extracted from the CaCO_3_ surface layer (fraction 1), intermediate layer (fraction 2) and the core region (fraction 3) of microparticles (Fig. 2a) and examined using TEM. We found that average particle sizes are similar (~3.7 nm) for all fractions (Figs 2c–e and S6). The sample obtained in a homogeneous solution of DS in water does not show the formation of particles (Fig. 2f) and is clearly different from those samples that have been extracted from CaCO_3_–DS microparticles.

Selected area electron diffraction (SAED) data (Figs 2b and S7) for all fractions show a number peaks that can be attributed to small crystallites of Ca(OH)_2_, possibly formed on a TEM grid while drying. All fractions show peaks corresponding to 2.1 Å (0.48 Å^−1^, close to the graphene sheet 100 reflection value 2.1 Å), while fraction 1 has a broad peak (marked with an star) at 2.40 Å (0.42 Å^−1^, close to 1120 reflection in graphite and graphene oxide at 2.46 Å) corresponding to the lattice planes observed in graphene and graphene oxide quantum dots^48^. Only fraction 3, obtained from the core of the CaCO_3_ particles, has the reflection that corresponds to the interlayer spacing in graphite (3.46 Å, 0.28 Å^−1^, 002 reflection, Fig. S8). One can also see that there is a much closer similarity between SAED patterns of fractions 2 and 3 than between them and fraction 1. CNDs prepared in water solution show amorphous-like diffraction pattern with no clear reflections. To aid further understanding of the atomic structure of samples we performed Raman measurements.

**Figure 3:**
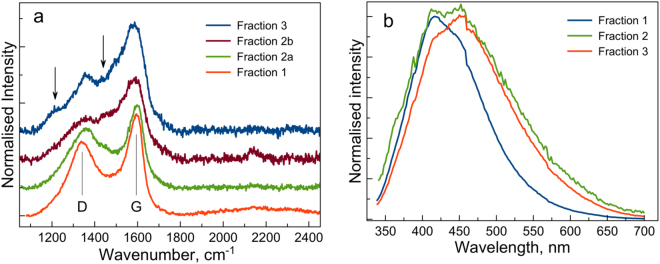
(**a**) Raman data for fractions 1, 2 and 3. Two different spectra (designated Fraction 2a and Fraction 2b) were observed for the fraction 2. Features at around 1200 cm^−1^ and 1450 cm^−1^ are marked with arrows. (**b**) Normalized PL data for all three fractions are also shown (exc. 320 nm).

In carbon systems Raman data can provide valuable information on the level of structural ordering owning to resonant excitations of π states. Raman can also be used to trace the variation from single layer, to graphite-like, through to nanocrystalline graphite and amorphous carbons and thus to evaluate the relative fractions of sp^2^ and sp^3^ bonded structures^49–53^. We found that Raman spectra for the three selected fractions are distinctively different (Fig. 3a). Samples extracted from the shell clearly show D band around 1350 cm^−1^ (K-point phonons of A_1g_ symmetry, defect band) and G band around 1580–1600 cm^−1^ (zone center phonons of E_2g_ symmetry). The G-band involves the in-plane bond-stretching motion of pairs of sp^2^-bonded C atoms and does not require the presence of six-fold rings. At the same time, the D band is a breathing mode that is forbidden in perfect graphite structures and only becomes active in the presence of disorder (including surface contribution in the case of small particles). Its intensity is proportional to the number of six-fold aromatic rings (for which the defect band is active), while broadening of D band reflects disorder in such clusters. Raman spectrum of fraction 1 demonstrates clearly distinguishable and relatively narrow D and G bands which are similar to the Raman signal observed in graphene oxide^53,54^. The broadening of D-band and gradual reduction in the relative intensity (i.e. I_D_/I_G_ ratio) of the D and G bands in the spectra of fractions 2 and 3 corresponds to increasing bond-angle disorder and reduction in the number of six-fold rings in the structure of these fractions^50^. We detected two types of spectra (designated as fraction 2a and 2b in Fig. 3a) for the fraction 2 with the spectral features showing gradual broadening of D and G bands and reduction in the relative intensity of the D band. This is the result of probing a larger sample volume compared to the TEM measurements and suggests that fraction 2 may be structurally intermediate between fractions 1 and 3. The gradual broadening of the G band is the consequence of growing disorder of sp^2^-bonded sites, while drop in G band intensity indicates reduction of relative proportion of sp^2^-bonded sites^50^ (i.e. increase in sp^3^-bonding character). Thus, although fractions 2 and 3 do also exhibit two major peaks associated with D and G bands (1354 cm^−1^ and 1590 cm^−1^), they are clearly more complex systems with broader peaks, while also showing additional features at around 1200 cm^−1^ and 1450 cm^−1^ (marked with arrows). The shoulder at around 1200 cm^−1^ is frequently observed in nanodiamonds^54,55^, but features in this range can also be associated^56^ with C-C stretching modes in hydrocarbon chains. The feature observed at around 1450 cm^−1^ can be assigned to CH_2_ scissoring mode^57^. Thus, we find that the Raman data are consistent with the SAED results showing more complex spectra for fractions 2 and 3 and hence a clear influence of the reaction volume configuration on CNDs’ structure. Furthermore, features of both fraction 1 and fraction 3 can be found in the fraction 2 (Fig. 3a).

This trend in variation of physical properties of CNDs across CaCO_3_ microparticles volume is also observed in PL spectra recorded for all three fractions (Fig. 3b). One can see a clear shift of the PL peak maximum for fractions 1 to 3, while fraction 2 contains both peaks. Interestingly, in our measurements this trend is observed for the PL data recorded in colloidal water suspension of CNDs where PL has been found to be governed by the surface states rather than by the carbon core^38^. This suggests variations in the surface structure across fractions, which is consistent with the Raman and SAED data.

Thus, experimental data clearly show a systematic variation of structure and light emission in CNDs prepared inside nanopores of CaCO_3_ microparticle with samples extracted from well-defined porous layers. Furthermore, we show that thus prepared CNDs are structurally different from those synthesized in DS solution in water where amorphous-like structure is observed. We believe that similarity of the Raman and TEM data for the obtained structures with those reported for CNDs prepared by hydrothermal synthesis route^23^ may suggest a similar formation mechanism: DS dehydration, followed by polymerization and finally formation of CNDs in CaCO_3_ pores that act as nanoscale reaction volumes. However, synthesis within the confined volume of nanoscale pores in CaCO_3_ microparticles allows to control precisely the amount of precursors and shows a clear effect on particle size and size distribution (see Fig. S6).

## Conclusions

In conclusion, we demonstrated a relatively simple method of synthesis of hydrophilic CNDs in pores of CaCO_3_ microparticles using DS as a carbon source. The results clearly show that light emission does not depend on initial concentration of DS in the 2–10 mg/ml range, while the restriction of the reaction volume can be used to influence CNDs structure and light emission and to avoid formation of large microparticles. TEM, SAED, Raman and PL data all point to the influence of pore morphology on the structural properties and light emission in CNDs. CNDs with graphene oxide like structure were obtained in the nanoporous outer shell layer of CaCO_3_ microparticles, while less ordered CNDs with the evidence of complex disordered carbons were extracted from the inner microcavity. These results suggest a very interesting direction for controlled CND synthesis whereby confinement in nanoscale pores can be used to control the amount of regents and reaction volume size, while the pore morphology (e.g. planar, 3D, linear) can be used to encourage formation of configurations with sp^2^ and sp^3^ hybridizations and thus to control the structure of the final product. At that, porous CaCO_3_ as a sacrificial template for CNDs synthesis can be a particularly promising route due to relatively simple precursor uptake and CND extraction.

## Methods

### Fabrication of CaCO_3_ microparticles

Uniform, nearly spherical CaCO_3_ microparticles with narrow size distribution were prepared by colloidal crystallization from supersaturated (relative to CaCO_3_) solution. The process was initiated by rapid mixing of equal volumes of Na_2_CO_3_ (Reakhim) и CaCl_2_ (CaCl_2_:2Н_2_О, Serva) solutions. In a typical experiment, equivalent volumes (0.615 ml) of 1 M Na_2_CO_3_ and CaCl_2_ solutions were rapidly poured into the solution of DS (DS, MW 40 kDa, Sigma, concentrations after dilution was 0, 2, 5, and 10 mg/ml) at room temperature. After intense agitation on a magnetic stirrer the precipitate was filtered off, thoroughly washed with bidistilled water and dried in air. The procedure results in highly homogeneous, spherical CaCO_3_ microparticles with an average diameter ranging from 4 to 6 µm. For hydrothermal treatment CaCO_3_-DS microparticles obtained in 12 parallel synthesis runs were collected together, dried and placed in a steel autoclave with teflon liner and a heat-resistant glass (Fig. S7). The autoclave was placed in a muffle furnace, heated up to 200 °C at 2.5 °C/min, held for 180 min at this temperature and then cooled. Additional experiments showed that CaCl_2_ and Na_2_CO_3_ precipitation leads to DS co-precipitation mainly into CaCO_3_ microparticles (Table S1). For control experiments, DS water solutions (4 ml) with concentrations of 2, 5 and 10 mg/ml were hydrothermally treated at the same conditions.

Following the thermal treatment several fractions of carbon material were collected starting from the surface layer towards the centre of CaCO_3_ microparticles. Surface fraction of CNDs was washed from the CaCO_3_ microparticles surface with 2 ml of water. CNDs formed inside CaCO_3_ microparticles pores, were extracted from CaCO_3_ using НСl solution (0.1 М), which was added step-by-step in fractions of 2 ml. Following each step of NaCl addition, the suspension was shaken, centrifuged (5000 rpm), and the supernatant (fraction) was removed. The process was repeated until the CaCO_3_ microparticles were completely dissolved. A number of fractions were broadly defined as a surface fraction (fraction 1, obtained from the surface layer), intermediate layer fraction (fraction2, extracted roughly half way towards the particle centre) and the core region (fraction 3, extracted from the core region). All synthesis runs were carried out 3 times and PL was measured. We found that the position of the PL maxima where the same within the experimental error while the intensity variation was no more than 5%.

### Experimental Characterization

Measurements of the absorption spectra was carried out using a Shimadzu UV-1800 spectrophotometer (Shimadzu, Japan). PL spectra and excitation-dependent PL were measured using a multifunction fluorimeter Cary Eclipse (Agilent Technologies, Australia).

SEM measurements to characterize the fabricated CaCO_3_-DS (shape, size, porosity and surface morphology) were performed using MIRA II LMU system (TESCAN, Czech Republic). Ultramicrotome Leica EM UC7 (Leica, Germany) was used to prepare the ultrathin sections of the CaCO_3_ microparticles to examine their internal structure. For SEM measurements, the water suspension of CaCO_3_ microparticles was drop-casted onto the silicon wafer and air-dried at room temperature. Prior to the SEM measurements the samples were sputtered with gold. Measurements were performed at operating voltages of 3–30 keV.

Light emission from as-prepared microparticles (before extraction of CNDs) was checked with Leica TCS SP8 X inverted confocal microscope (Leica Microsystems) using Leica 100x/1.44 NA oil N-PLAN objective. The PL was excited by 405 nm laser and the emission λ-scan was recorded from 410 to 750 nm.

Renishaw inVia (Renishaw, UK) Raman confocal microscope equipped with 532 nm and 785 nm lasers was used to acquire the Raman data. Samples were pipetted on gold layer evaporated on Si substrate. The laser beam was focused through a 50x(Leica N PLAN L, NA 0.5) microscope objective and the Raman data were collected in the backscattering mode.

Transmission electron microscopy (TEM) was performed using a JEOL 2010 transmission electron microscope (JEOL, Japan) operated at 200 kV. The system resolution was around 0.2 nm. For the TEM studies, samples in suspension were drop-casted on an ultrathin amorphous carbon film supported on a Cu grid and dried in air.

## Electronic supplementary material

Supplementary Information
