# Supplementary material for: Thermal carbonization in nanoscale reactors: controlled formation of carbon nanodots inside porous CaCO3 microparticles
Source: Sci Rep. 2018 Jun 20;8:9394. doi: 10.1038/s41598-018-27488-w (PMC6010419; doi:10.1038/s41598-018-27488-w)

**Supporting information**

Thermal carbonization in nanoscale reactors: controlled formation of carbon nanodots inside porous CaCO_3_ microparticles

Anna V. Vostrikova,^a^ Ekaterina S. Prikhozhdenko,^a^ Oksana A. Mayorova,^a^ Irina Yu. Goryacheva,^a^ Nadezda V. Tarakina,^b^ Gleb B. Sukhorukov,^а,b^ Andrei V. Sapelkin ^а,c^

^a^SARATOV STATE UNIVERSITY, 83 Astrakhanskaya Street, Saratov, 410012, Russia

^b^SCHOOL OF ENGINEERING AND MATERIALS SCIENCE, QUEEN MARY UNIVERSITY OF LONDON, Mile End Road, E1 4NS, UK

^c^SCHOOL OF PHYSICS AND ASTRONOMY, QUEEN MARY UNIVERSITY OF LONDON, Mile End Road, E1 4NS, UK


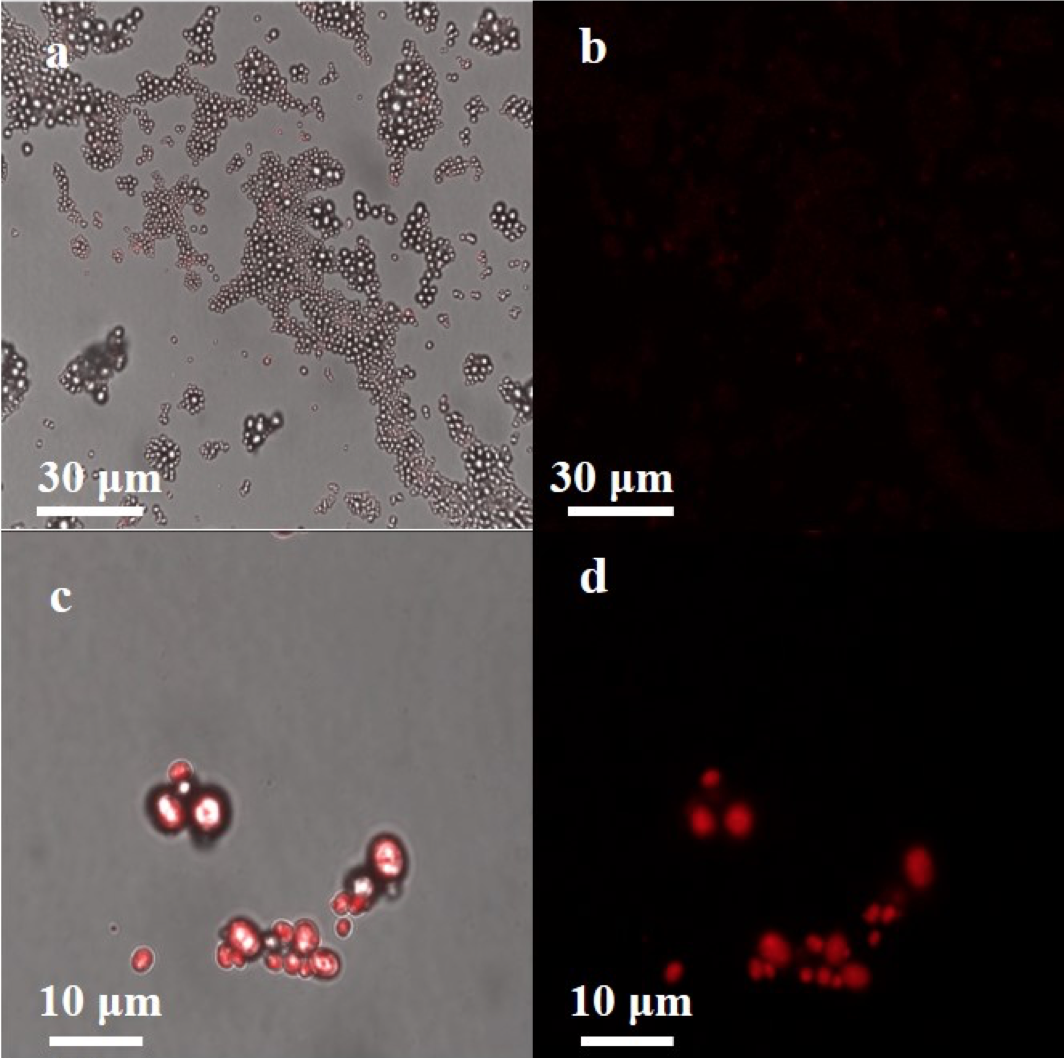


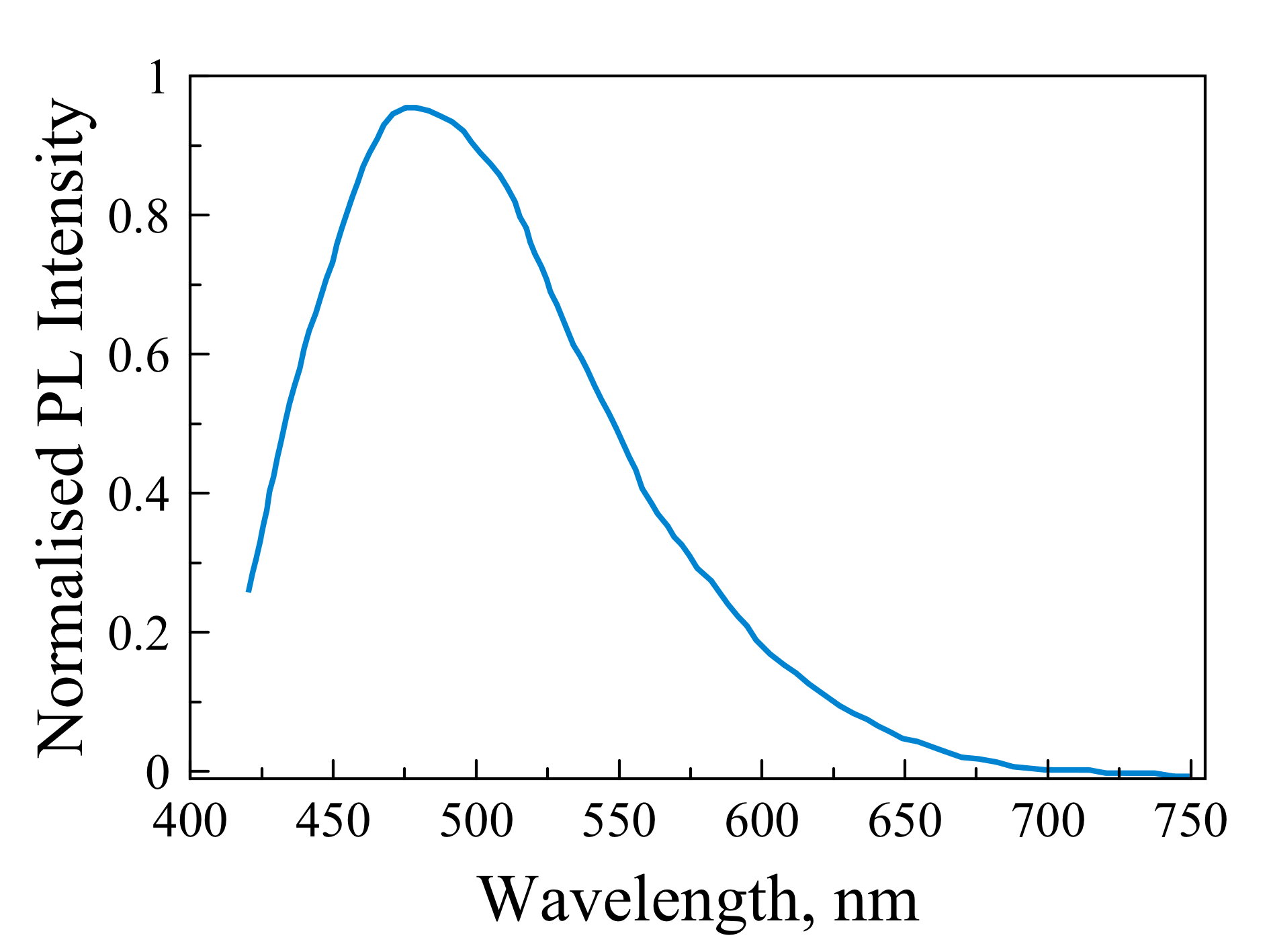


e

**Figure. S1** Confocal images (λ_ex_ = 405 nm) of CaCO_3_–DS microparticles before (a, b) and after (c, d) hydrothermal treatment; normalized PL spectra of hydrothermally-treated CaCO_3_–DS microparticles obtained by CLSM λ-scan detection (λ_ex_=405 nm), (e).

**DS inclusion into CaCO_3_ microparticles**


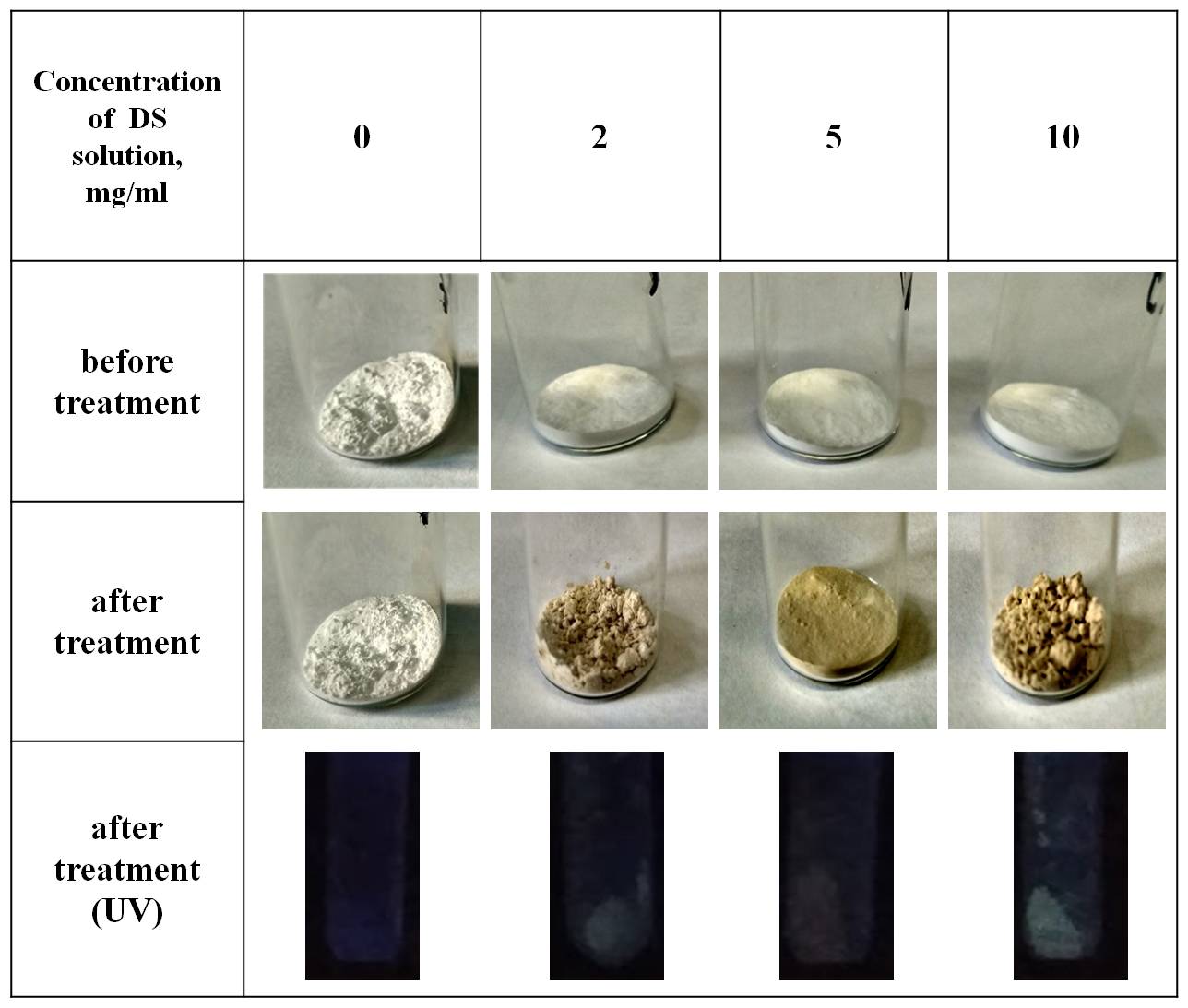


**Figure S2.** CaCO_3_–DS microparticles before and after thermal treatment and under UV (365 nm) excitation.


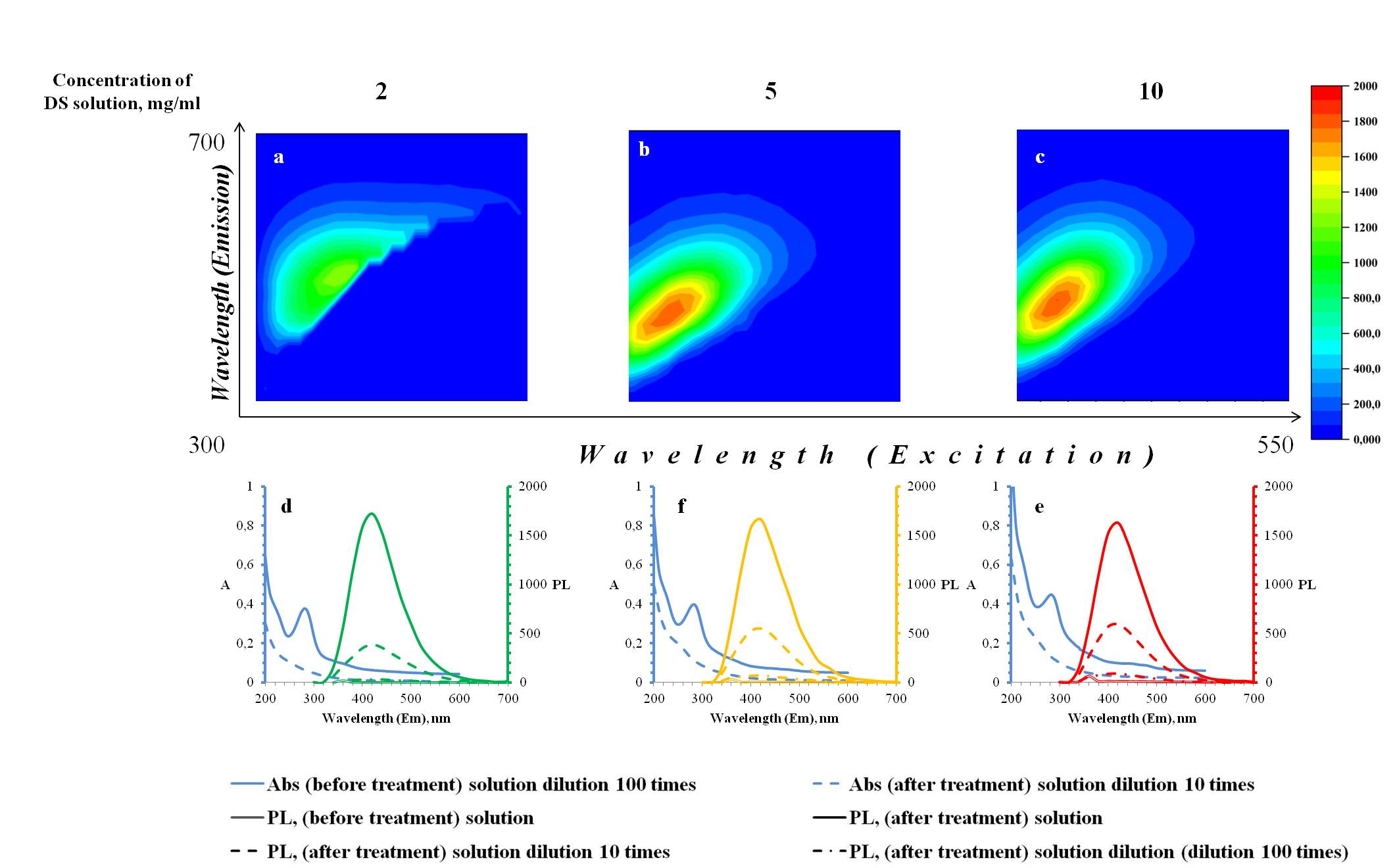


**Figure S3.** 3D and PL spectra of supernatants obtained after washing of thermally-treated CaCO_3_–DS microparticles (λ_ex_ = 320 nm). Initial DS solution concentrations were 2 mg/ml (a, d), 5 mg/ml (b, e), 10 mg/ml (c, f). Spectra from diluted samples presented as dashed lines (λ_ex_=320 nm).


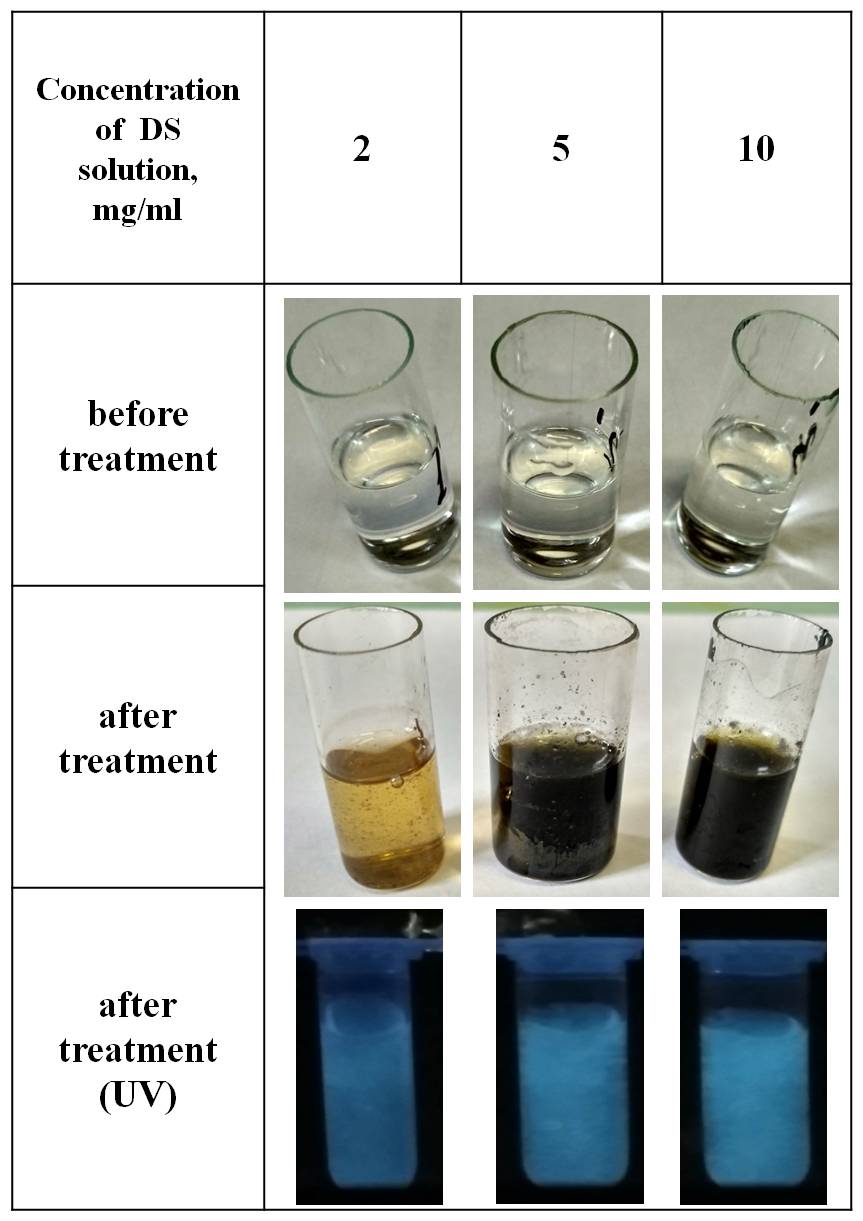


**Figure S4.** DS solution before (A) and after thermal treatment (B) and under UV (365 nm) excitation (C).


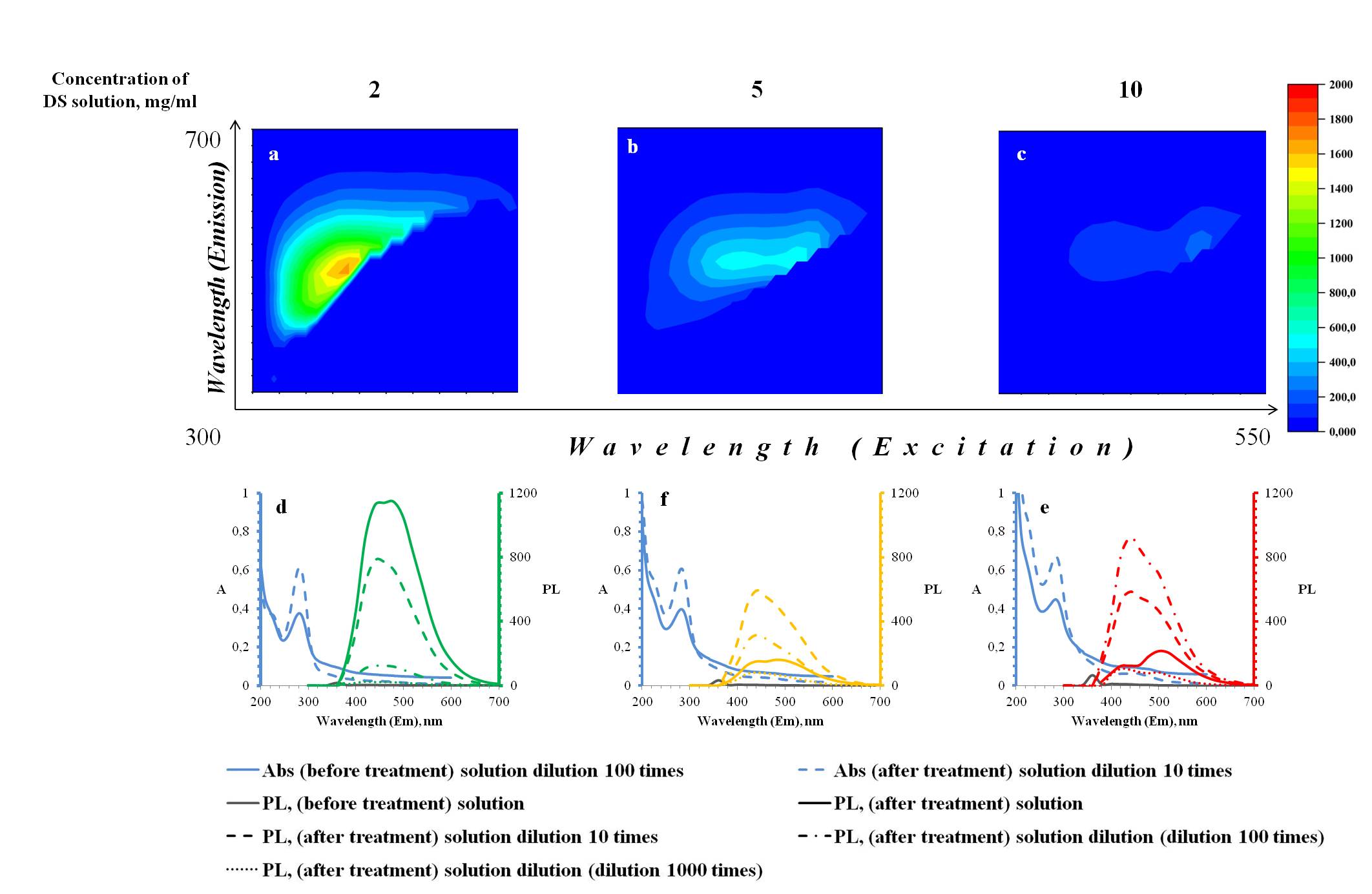


**Figure S5.** PL spectra and corresponding excitation-emission maps of CNDs obtained after hydrotermal treatment of DS solutions 2 mg/ml (a, d), 5 mg/ml (b, e), 10 mg/ml (c, f). Spectra from diluted samples presented as dashed lines (λ_ex_ = 320 nm).


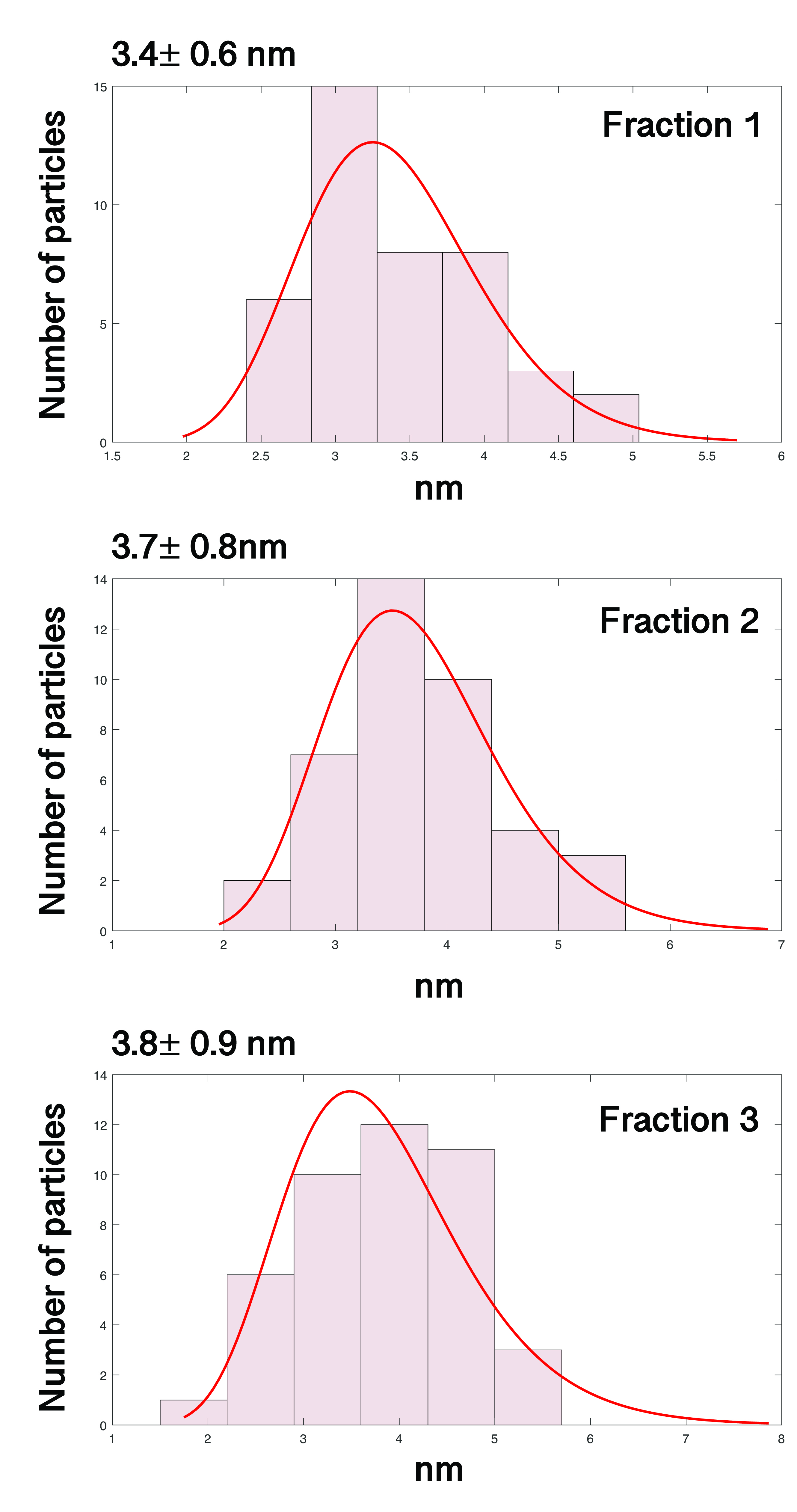


**Figure S6.** CNDs particle size distribution histograms obtained from TEM images of 3 different fractions with log-normal distribution fits (solid lines). The uncertainties shown are those of the fitting a log-normal distribution to the corresponding histograms. The experimental TEM resolution was estimated to be around 0.2 nm.


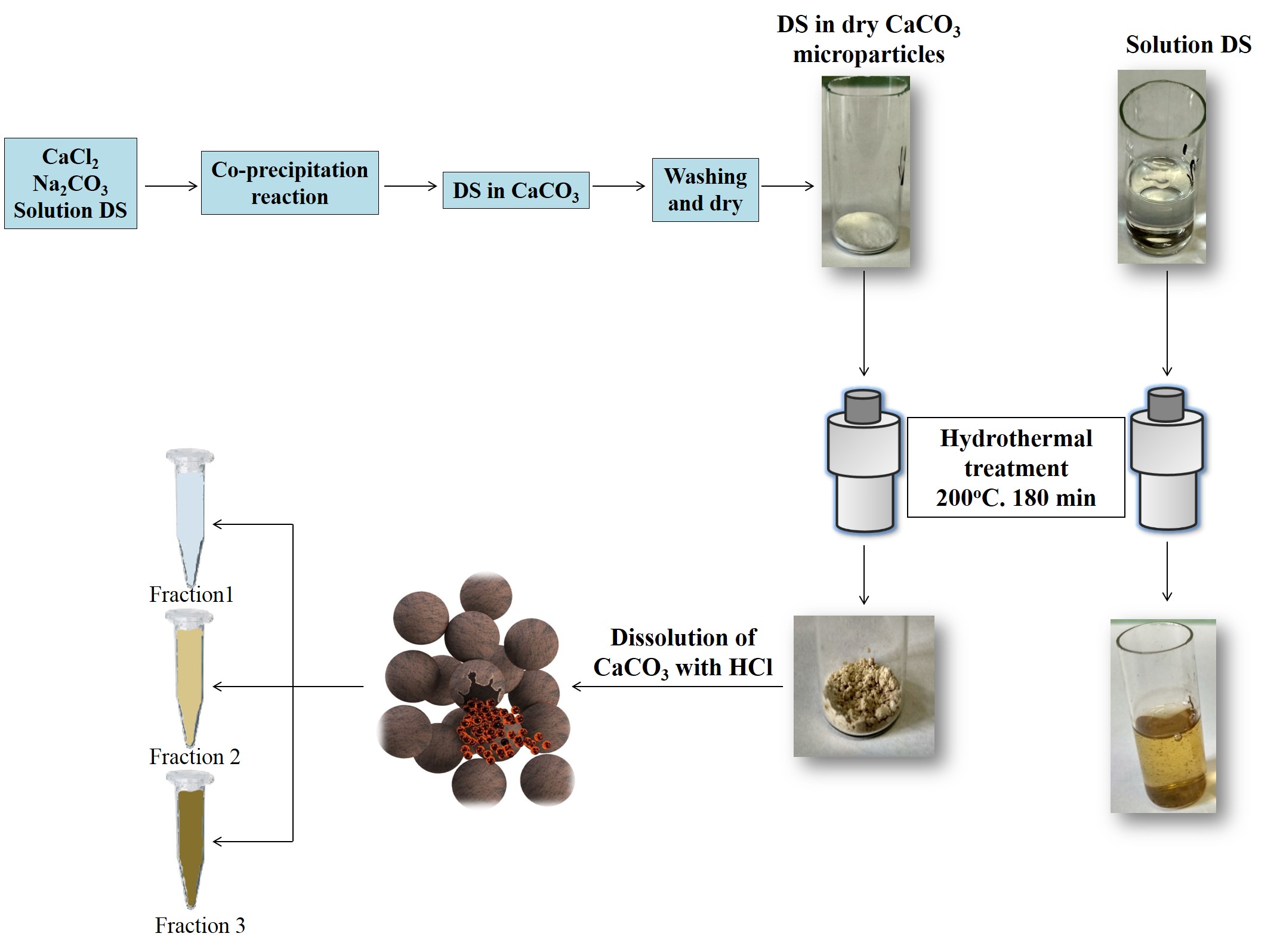


**Figure S7.** Schematic diagram of CNDs preparation

_
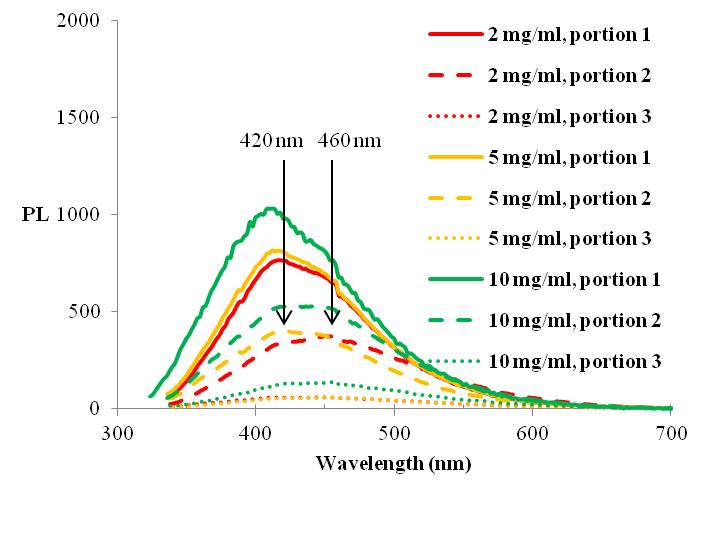
_

**Figure S8.** PL spectra of portions 1 (solid line), 3 (dashed lines) and the last one (dotted line) (λ_ex_ = 320 nm) for different DS initial concentrations: 2 (red), 5 (yellow) and 10 (green) mg/ml.

**Table S1**. For examination of DS inclusion into CaCO_3_ microparticles two experiments were performed: with and without DS (the latter was used as a control). CaCO_3_ and CaCO_3_-DS microparticles were dried, weighted and difference with and without DS was calculated.


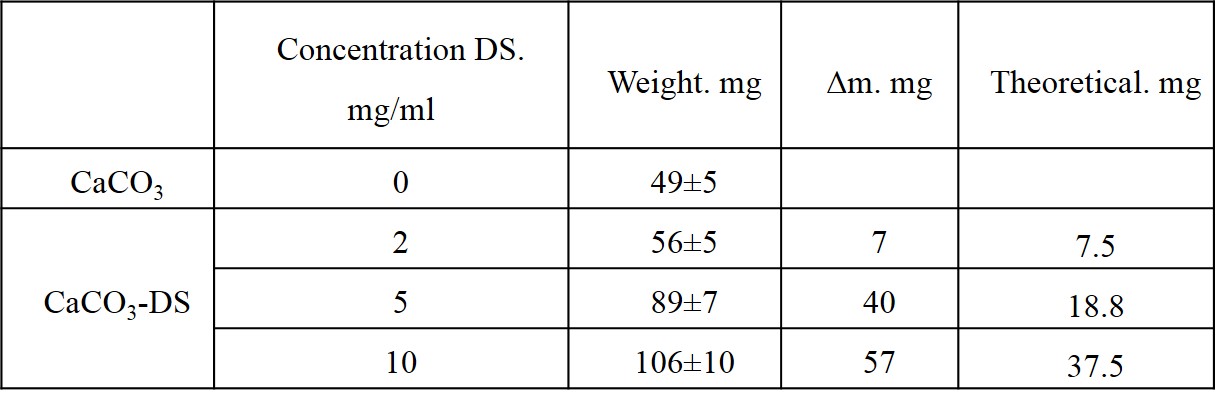

Supplement: Supplementary file 1 — Supplementary Information [file 41598_2018_27488_MOESM1_ESM.docx]
